# Cerebral arteriopathy associated with heterozygous variants in the casitas B-lineage lymphoma gene

**DOI:** 10.1212/NXG.0000000000000448

**Published:** 2020-06-10

**Authors:** Ying Hong, Annette Keylock, Barbara Jensen, Thomas S. Jacques, Olumide Ogunbiyi, Ebun Omoyinmi, Dawn Saunders, Andrew A. Mallick, Madeleine Tooley, Ruth Newbury-Ecob, Julia Rankin, Hywel J. Williams, Vijeya Ganesan, Paul A. Brogan, Despina Eleftheriou

**Affiliations:** From the UCL Great Ormond Street Institute of Child Health (Y.H., A.K., B.J., T.S.J., E.O., D.S., V.G., P.A.B., D.E.); Histopathology Department (O.O.), Great Ormond Street Hospital, London; Paediatric Neurology Department (A.A.M.), and Genetics Department (M.T., R.N.-E.), Bristol Royal Hospital for Children; Genetics Department (J.R.), Royal Devon and Exeter NHS Foundation Trust, Exeter; Centre for Translational Omics–GOSgene (H.J.W.), UCL GOS Institute of Child Health; and Centre for Adolescent Rheumatology Versus Arthritis (D.E.), London, United Kingdom.

## Abstract

**Objective:**

To report a series of patients with cerebral arteriopathy associated with heterozygous variants in the casitas B-lineage lymphoma (*CBL*) gene and examine the functional role of the identified mutant Cbl protein. We hypothesized that mutated Cbl fails to act as a negative regulator of the RAS-mitogen-activated protein kinases (MAPK) signaling pathway, resulting in enhanced vascular fibroblast proliferation and migration and enhanced angiogenesis and collateral vessel formation.

**Methods:**

We performed whole-exome sequencing in 11 separate families referred to Great Ormond Street Hospital, London, with suspected genetic cause for clinical presentation with severe progressive cerebral arteriopathy.

**Results:**

We identified heterozygous variants in the *CBL* gene in 5 affected cases from 3 families. We show that impaired *CBL*-mediated degradation of cell surface tyrosine kinase receptors and dysregulated intracellular signaling through the RAS-MAPK pathway contribute to the pathogenesis of the observed arteriopathy. Mutated *CBL* failed to control the angiogenic signal relay of vascular endothelial growth factor receptor 2, leading to prolonged tyrosine kinase signaling, thus driving angiogenesis and collateral vessel formation. Mutant Cbl promoted myofibroblast migration and proliferation contributing to vascular occlusive disease; these effects were abrogated following treatment with a RAF-RAS-MAPK pathway inhibitor.

**Conclusions:**

We provide a possible mechanism for the arteriopathy associated with heterozygous *CBL* variants. Identification of the key role for the RAS-MAPK pathway in *CBL*-mediated cerebral arteriopathy could facilitate identification of novel or repurposed druggable targets for treating these patients and may also provide therapeutic clues for other cerebral arteriopathies.

The casitas B-lineage lymphoma (*CBL*) gene encodes a member of the Cbl family of proteins that function as a RING finger E3 ubiquitin ligase.^1^ This Cbl protein has an N-terminal phosphotyrosine binding domain that interacts with tyrosine-phosphorylated substrates to target them for proteasome degradation.^2^ Through this process, the Cbl protein acts as a negative regulator of signal transduction pathways, such as the RAS-mitogen-activated protein kinases (MAPK) signaling pathway.^3^ Previous reports have identified that missense mutations in *CBL* cause a disorder characterized by impaired growth, cryptorchidism, predisposition to juvenile myelomonocytic leukemia, and systemic arteriopathy in some cases.^4–7^ Isolated cases of cerebrovascular disease have also been reported in association with heterozygous de novo mutations in *CBL*.^4,6^ The exact mechanism of the observed arteriopathy remains unclear, however. Here, we identified heterozygous *CBL* variants in 5 individuals with cerebral arteriopathy from 3 separate families (figure 1, A–C) and for the first time examined the functional role of mutant Cbl protein in this context. We hypothesized that mutated Cbl fails to act as a negative regulator of the RAS-MAPK signaling pathway, resulting in enhanced vascular fibroblast proliferation, migration, and enhanced angiogenesis and collateral vessel formation.

**Figure 1:**
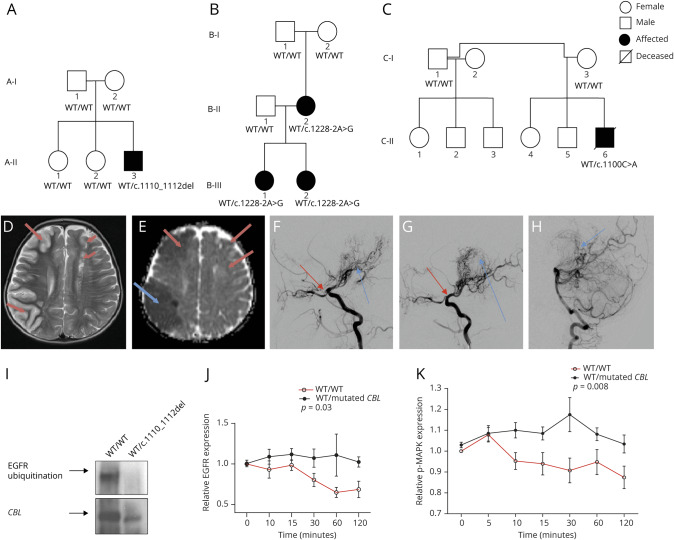
Loss of CBL-mediated ubiquitination activity in mutant CBL cells results in reduced Cbl-regulated cell surface receptor degradation and enhanced cellular tyrosine kinase signaling (A–C) Pedigrees for all 3 studied families show the affected and unaffected family members; segregation of *CBL* variants is also shown where genetic sequencing was available. (D and E) Axial T2 and apparent diffusion coefficient map from the diffusion-weighted imaging for A-II-3 showed an acute right largely cortically based right middle cerebral artery (MCA) territory infarct (long blue arrow). Mature deep and cortical left anterior cerebral artery (ACA)/MCA watershed infarcts indicate previous left hemispheric injury (short red arrows). (F–H) Catheter digital subtraction angiography for A-II-3 showed a right internal carotid artery (ICA) stenosis with some filling of a narrow right MCA branch and occluded right ACA (red arrow) and multiple moyamoya, ethmoidal, and ophthalmic collateral vessels (blue arrow). (G) The left ICA was occluded just beyond the left PCA (red arrow) and ACA branch can be seen to fill via collaterals. The collateral pattern is similar to the right with additional pial collaterals from the left PCA (blue arrow). (H) The posterior circulation vessels were normal and fill the cerebral hemispheres via collateral pial vessels (arrow). (I) Peripheral blood mononuclear cells (PBMCs) from patients with heterozygous mutations in CBL (B-III-1 and B-III-2) were lysed and immunoblotted with an anti-CBL antibody, followed by a ubiquitination assay using a known Cbl substrate, epidermal growth factor (EGFR), and compared with control cells. The blot shown here indicates impaired Cbl-mediated EGFR ubiquitination in PBMCs with mutated Cbl. (J) PBMCs from patients with heterozygous *CBL* mutations were stimulated with 20 ng/mL EGF for the indicated periods of time and stained to explore changes in EGFR expression. Relative EGFR expression was increased for patient-derived cells at all time points compared with EGFR expression for healthy control cells, *p* = 0.03. (K) Similarly, there was increased phosphorylation of MAPK in patient PBMCs compared with control cells at all time points, *p* = 0.008. Data were expressed as fold change relative to mean baseline expression for control and plotted as mean of triplicate samples ± SEM. *p* <0.05 calculated by analysis of variance and unpaired *t* tests were considered significant. CBL = casitas B-lineage lymphoma; MAPK = mitogen-activated protein kinase.

## Methods

### Standard protocol approvals, registrations, and patient consents

This study was approved by the Bloomsbury ethics committee (ethics number 08H071382). We obtained written informed consent from all the family members and controls who participated, and additionally for children, written assent where appropriate.

### Patients

We screened 11 families where the index case was presenting with suspected genetic cerebral arteriopathy due to disease onset from early in life, other affected family members, or presence of dysmorphic features. All cases were referred for specialist opinion to the vasculitis and neurovascular service at Great Ormond Street Hospital, London, between October 2016 and January 2019. Ischemic strokes were diagnosed on the basis of the presence of cerebral infarct on confirmatory cerebral imaging or diagnosis of a TIA.^8^ Cerebral/cervical arteriopathy was defined as focal or segmental arterial stenosis or occlusion, with regular or irregular abnormalities of the arterial wall and categorized according to current consensus definitions.^8,9^ Diagnosis of moyamoya arteriopathy was based on cerebrovascular imaging demonstrating stenosis or occlusion of the terminal portion of the internal carotid artery, the formation of an abnormal vascular network in the vicinity of the arterial occlusion, and review of the patient's history and images for exclusion of other causes of arterial stenosis.^8,9^ In vitro studies were performed in patient- and age-sex–matched healthy control–derived peripheral blood mononuclear cells (PBMCs), cultured dermal fibroblast cells, transformed smooth muscle cell (SMC)-like cells, human brain vascular adventitial fibroblasts (HBVAFs; ScienCell, Carlsbad, CA), and human umbilical vein endothelial cells (HUVECs; PromoCell, Heidelberg, Germany).^10,11^

### Genetic mapping and sequencing

Trios were genotyped using the Illumina cytoSNP12 chip. A total of 200 ng of DNA was isothermally amplified overnight and then enzymatically fragmented. The fragmented DNA was incubated on a BeadChip overnight. These were imaged using the Illumina iScan Read. Whole-exome sequencing was completed using the Illumina and sequenced on the Illumina HiSeq2000. The raw sequence data were aligned to the human reference genome using the Burrows-Wheeler Aligner alignment algorithm. Variant calling was with the Genome Analysis ToolKit. Variant annotation was with wANNOVAR. Annotated data from whole-exome sequencing were screened in the first instance for rare novel or rare nonsynonymous variants (<0.01) in the 1000 Genomes Project and Genome Aggregation Database. For every rare variant, we assessed pathogenicity, novelty, and predicted functional impact using SIFT, PolyPhen-2, MutationTaster, and Alamut Batch version 2.11. The identified variants were individually assessed and classified into pathogenicity groups (Class 1: clearly not pathogenic; Class 2: unlikely to be pathogenic; Class 3: unknown significance; Class 4: likely to be pathogenic; and Class 5: clearly pathogenic), according to the Association of Clinical Genetics Science Practice Guidelines 2013 guidelines.^12^ Variant confirmation and familial segregation were ascertained by PCR, and sequencing was completed with the AB3730 using the BigDye v3.1 kit (Applied Biosystems, Foster City, CA).

### Cbl-induced ubiquitination assay

PBMCs were isolated from heparinized blood using Ficoll separation. Cbl ubiquitination activity was measured using the Ubiquitylation Assay Kit (Abcam, Cambridge, UK). Protein was extracted from PBMCs using M-PER mammalian protein extraction reagent (Thermo Fisher Scientific, Waltham, MA), supplemented with protease inhibitor cocktail. A total of 150 mg of whole cell lysate was incubated with rabbit anti-Cbl (Santa Cruz) and with Protein A agarose beads (Santa Cruz, Dallas, TX). Washed Cbl immunoprecipitates were used for ubiquitination assay, as per manufacturer instructions.

### Fibroblast culture and myofibroblast trans-differentiation

Human dermal fibroblast cells (HDFCs) were explanted from patients heterozygous for *CBL* mutations and from controls without any identifiable vascular disease. HDFCs and commercially available HBVAFs were cultured in basal medium (Ham F10 Nutrient Mix). For trans-differentiation, HDFCs were evenly seeded on matrigel precoated plates. After overnight incubation, cells were treated with 2% heat-inactivated horse serum and 5 ng/mL transforming growth factor β1 to induce fibroblast trans-differentiation into SMC-like cells. The cells were harvested on day 14 for RNA isolation. qPCR analysis was performed to analyze mRNA expression of an SMC marker gene (ACTA2). The migratory potential of these cells was assessed with a wound healing scratch assay. Fibroblasts were cultured until confluency in 6-well culture plates, as described above. A scratch was made in the confluent monolayer with a plastic disposable pipette tip (200 μL). Cultures were washed twice with PBS to remove all detached cells. The wound area was photographed at *t* = 0 and *t* = 24 hours with a phase contrast microscope. The migrated area was determined by subtracting the wound area at these time point and cells migrating in the area counted.

### Matrigel assays: HUVEC capillary networks

HUVECs were cultured in EGM-2 medium supplemented with 2% fetal calf serum, hydrocortisone, fibroblast growth factor 2, vascular endothelial growth factor (VEGF), R3-insulin-like growth factor 1, epidermal growth factor, heparin, ascorbic acid, gentamycin, and amphotericin B, as supplied by the manufacturer (PromoCell) at 37°C in 5% CO_2_ in a humidified incubator. HUVECs at 80% confluency (passages 2–5) were used for experiments. Matrigel assays were used to examine the ability of HUVECs to form endothelial capillary networks.^8^ Growth factor–reduced Matrigel matrix (Becton Dickinson Labware, Oxford, UK) was thawed and placed in 96-well plates at 37°C for 30 minutes to solidify. HUVECs (1 × 10^4^ cells/well) were seeded onto polymerized Matrigel and incubated in EGM-2 medium at 37°C for 16 hours. The cultures were photographed with an Olympus TH4-200 microscope. Capillary-like tube networks were observed, and the branch point number of 5 independent fields each well was counted.^10^

### Flow cytometric analysis of EGFR, phosphorylated MAPK, PLC-γ1, Ki67 expression in PBMCs, HUVECs and HDFCs and SMC myofibroblasts

Before stimulation, HUVECs, HDFCs, HBVAFs, and SMC myofibroblasts were washed with sterile PBS and put in quiescent (basal) media for 24 hours. PBMCs were left in normal media overnight after being extracted or thawed. HUVECs were stimulated with 20 ng/mL recombinant human VEGF 165 protein (R&D Systems, Minneapolis, MN); PBMCs were stimulated with 20 ng/mL epidermal growth factor (EGF) recombinant human protein (Thermo Fisher Scientific), and transformed HDFCs were stimulated with 20 ng/mL platelet-derived growth factor (PDGF) A/B (PeproTech, Rocky Hill, NJ). To detect the expression of epidermal growth factor receptor (EGFR), PBMCs were fixed and permeabilized using BD Cytofix/CytopermTM after stimulation with EGF. Cells were stained with a monoclonal antibody against EGFR (Santa Cruz), followed by a secondary antibody, a rabbit anti-mouse Alexa 488 (Life Technologies, Carlsbad, CA). To detect phosphorylated MAPK expression in PBMCs, HDFCs, HBVAFs, and SMC myofibroblasts and phosphorylated PLC-γ1 expression on HUVECs, following the stimulations, cells were fixed in prewarmed BD Cytofix buffer for 10 minutes at 37°C. Cells were then washed and permeabilized with BD Phosflow Perm Buffer III for 30 minutes at 40°C. After cells were incubated with the primary antibody for p-MAPK or p-PLC-γ1 (Cell Signaling Technology, Danvers, MA) for 60 minutes on ice, cells were washed and stained with the secondary antibody, goat anti-rabbit Alexa 488 (Life Technologies) for further 45 minutes on ice. To assess Ki67 expression, resting or stimulated (with growth factor) cells were stained with FITC-conjugated anti-Ki67 (1:50 dilution; Miltenyi Biotec, Bergisch Gladbach, Germany) antibody. The expression of EGFR, p-MAPK, and phospholipase C-γ1 (PLC-γ1) and Ki67 was detected by flow cytometry analysis using an FACScalibur flow cytometer (BD Biosciences, Franklin Lakes, NJ).

### RAF-RAS-MAPK inhibitor effect on fibroblast proliferation and migration

Fibroblasts were pretreated with 5 μM of sorafenib, a protein kinase inhibitor that targets the RAF-RAS-MAPK pathway, for 30 minutes before stimulation with 20 ng/mL PDGF, and their migratory and proliferative capacity was then assessed.

### siRNA CBL transfection in fibroblasts and HUVECs

siRNA knockdown was performed in primary fibroblasts (HDFCs and HBVAFs) or HUVECs via transient transfection using with 100 nM of siRNA targeting *CBL* (SC-29242; Santa Cruz) or a negative control. siRNAs (SC-37007; Santa Cruz) were transfected via complexation with Lipofectamine RNAiMAX (13778-075; Life Technologies) according to the manufacturer's instructions. At 48 hours post-transfection, knockdown efficiency was evaluated by quantitative real-time PCR. The QuantiTect CBL primers were used (QT00070301; Qiagen, Hilden, Germany).

### Histopathology

Immunohistochemistry was performed using Phospho-p44/42 MAPK (Erk1/2) (Thr202/Tyr204) (D13.14.4E) XP Rabbit Monoclonal Primary Antibody (Catalogue No. 4370s) (Cell Signaling Technology, Beverly, MA), diluted at 1:50 on a Leica Bond-Max autostainer (Leica Biosystems, Melbourne, Australia) and the Leica Bond Polymer Refine Detection kit (Reference No. DS9800). Antigen retrieval was by heat-induced epitope retrieval, Leica Epitope Retrieval protocol 1 for 20 minutes, pH 6. The tissue sections were counterstained with hematoxylin.

### Data availability

The authors confirm that the data supporting the findings of this study are available within the article.

### Statistical analyses

Results were independently reviewed by 2 scientists (A.K. and Y.H.). Results were expressed as mean and SEM or median and range. All statistics (repeated-measures analysis of variance and unpaired Student *t* tests) were produced using Prism version 8 (GraphPad, San Diego, CA). 95% confidence intervals (CIs) for the difference of the means between groups are also provided when a *t* test was performed. *p* <0.05 (2 sided) was considered significant.

## Results

### Clinical features

A-II-3 was a 2.5-year-old male of Filipino nonconsanguineous descent (figure 1A) who presented with left hemiparesis. Brain MRI revealed an acute right middle cerebral artery (MCA) territory infarct (figure 1, D and E) with an established left-sided infarct in the periventricular white matter. Brain MRA and catheter angiography revealed bilateral occlusive arteriopathy involving the internal carotid artery (ICA), with basal collaterals (figure 1, F–H). Of note, visceral digital subtraction angiography and echocardiography were normal. There was no acute phase response (ESR 5 mm/h; reference range <10 mm/h; CRP 5 mg/L; reference range <20 mg/L); antineutrophil cytoplasmic antibody and antinuclear antibody testing was negative. Other metabolic and infectious disease investigations, including CSF analyses, were negative. As he was noted to have 2 café-au-lait spots, genetic screening of *NF1* gene mutations was undertaken through routine clinical laboratory testing, which excluded any pathogenic variants. In addition, array comparative genomic hybridization did not detect any copy number variants. He was also identified to have factor XII deficiency (factor XII levels 32 IU/L; normal range 50–150 IU/L), but did not experience any bleeding episodes, and had no evidence of intracerebral hemorrhage on radiologic imaging. He was started on aspirin (5 mg/kg/d), and at age 4 years, he underwent bilateral pial synangiosis. He has not had any further neurologic events.

**Figure 2:**
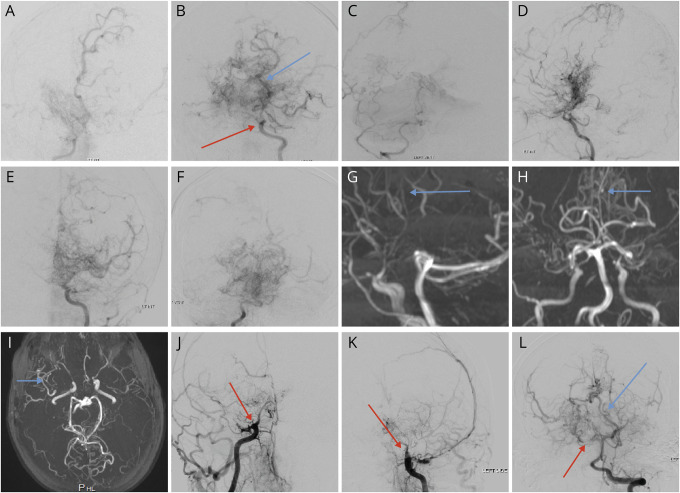
Cerebral and MRI angiography findings in patients with heterozygous CBL mutations and cerebral arteriopathy (A–C) Catheter angiogram (CA) for B-III-2 demonstrated a small and poorly filling right internal carotid artery (RICA), stenosed at the skull base (red arrow) and occluded at cavernous segment. The hemisphere was filled via moyamoya collaterals (red arrow) and a fetal right posterior cerebral artery (PCA). The left terminal internal carotid was severely stenosed and filled only a stenosed left middle cerebral artery (MCA) and multiple moyamoya collaterals. The right vertebral artery (VA) and basilar artery were small and did not fill the PCAs. (D–F) CA images for B-III-1 are shown. The RICA was occluded its cavernous segment with filling of some vessels via multiple moyamoya collaterals. The left ICA was occluded at the cavernous segment with severe stenosis of proximal anterior cerebral artery (ACA) and MCA and multiple moyamoya collaterals. The left VA was occluded, and the right VA is stenosed shortly before forming the basilar artery, which is small and which was occluded at it terminus with no filling of the PCAs. Multiple pial collaterals were visible. (G–I) (blue arrows) MRA images for B-II-2 revealed bilateral occlusion of the terminal ICAs (red arrows) with filling of distal vessels via moyamoya collaterals. The posterior circulation appeared patent but attenuated with pial collateral formation. (J–L) CA for C-II-6 revealed bilateral occlusion of the terminal ICAs with no distal filling via normal intracranial vasculature but some filling of the hemispheres from external carotid–internal carotid and skull base collaterals. The P1 segment of the right PCA was occluded (red arrow), but multiple pial collaterals were seen filling the hemispheres (blue arrow). CBL = casitas B-lineage lymphoma.

B-III-2 was a 4-month-old female of Caucasian nonconsanguineous descent (figures 1B and 2, A–F) who presented with an encephalopathy and left-sided focal seizures in the context of acute dehydration. She had a history of pulmonary valve stenosis diagnosed at birth, which had been surgically corrected. Brain MRI revealed acute swelling and signal change with restricted diffusion of the whole right hemisphere, but sparing the basal ganglia. Subsequent brain MRA and catheter angiography of the cerebral circulation showed an occlusive arteriopathy of the terminal ICAs, the MCA, and the anterior cerebral arteries. Computerized brain CT showed no intracerebral calcification. There were no systemic symptoms. There was no acute phase response (ESR 7 mm/h; reference range <10 mm/h and CRP 6 mg/L; reference range <20 mg/L); antineutrophil cytoplasmic antibody and antinuclear antibody testing was negative. Visceral angiography was normal. Extensive screening for infectious, metabolic, and other rheumatologic causes was negative. She was started on aspirin (5 mg/kg/d). She remained stable with no stroke recurrence, but developed progressive white matter disease in the left hemisphere over time. Intriguingly, her sister (B-III-1) also had a history of learning difficulties and was identified to have a similar pattern of arteriopathy on brain vascular imaging (figure 2, D–F), Her mother (B-II-2) had a history of dyspraxia and sensorineural hearing loss, but no clinical history suggestive of stroke. Brain MRI showed signal abnormality in the left mid-pons of uncertain significance, no evidence of acute ischemic stroke, and vascular imaging revealed the same pattern of arteriopathy (figure 2, G–I). Both affected siblings have undergone revascularization procedures: B-III-1 had multiple burr hole surgery, and B-III-2 had a left-sided encephaloduroarteriosynangiosis.

C-II-6 (figure 2, K–M), of Caucasian, nonconsanguineous descent, was born with an uncomplicated delivery, but was noted to be floppy in the first year of life, with gross and fine motor delay. During pediatric review at age 7 months for treatment of pneumonia, he was found to have hepatosplenomegaly. Despite numerous investigations at that stage, no clear cause was identified for his delayed development and hepatosplenomegaly. Over the years, he was noted to have down-slanting palpebral fissures, full lips, and progressive lymphedema. At age 29 years, he presented acutely with left-sided focal seizures and left hemiparesis and was subsequently found to have an acute right-sided intraparenchymal hemorrhage, with ventricular extension on brain MRI, and occlusive arteriopathy of the terminal ICAs with collaterals on angiography. He died despite neurosurgical intervention. Array comparative genomic hybridization did not find any copy number variants, and thus, further genetic testing was requested.

### Genetic sequencing

Whole-exome sequencing (WES) in A-II-3 revealed a novel variant, NM_005188:c.1110_1112del (p.L370_Y371delinsF), in *CBL*, a non-frameshift deletion of 3 nucleotides (ATA) resulting in the loss of 2 residues (a leucine and a tyrosine) and to the insertion of phenylalanine in the linker region of Cbl. The linker region contains 2 conserved tyrosine residues, Tyr-368 and Tyr-371, whose phosphorylation activates and positively regulates E3 ligase activity of Cbl. This variant was not detected in his unaffected siblings and parents.

WES of B-III-1, B-II-2, and B-III-2 revealed an intronic mutation in CBL: NM_005188:c.1228-2A>G in *CBL* present in the affected siblings and their mother and absent in the father (B-II-1) and maternal grandparents (B-I-1 and B-I-2). This variant was predicted to affect splicing and occurs upstream of exon 9, which encodes part of the RING finger domain of Cbl.

C-II-6 had a rare heterozygous missense mutation in *CBL*, identified posthumously: NM_005188:c.1100C>A (p.Q367P). This residue is located in the linker region of Cbl.

All variants detected using WES were confirmed with Sanger sequencing. In silico predictive tools (SIFT, MutationTaster, and PolyPhen-2) predicted that all these variants were likely to be damaging. Screening of the WES data sets for any other known genetic variants associated with cerebral arteriopathy did not identify any other pertinent Class 4 or Class 5 variants or any other variants in candidate genes of interest.

### Cbl-mediated ubiquitination activity in PBMCs from patients with heterozygous CBL variants

To determine whether mutant Cbl protein retains E3 ligase activity, we assessed ubiquitination of known Cbl substrate in patient-derived PBMCs. Cbl is ubiquitously expressed and negatively regulates various receptor protein tyrosine kinase signaling pathways via receptor degradation, thus acting as an adaptor protein in tyrosine phosphorylation-dependent signaling.^13^ In PBMCs, EGFR is one such receptor protein tyrosine kinase that soon after activation undergoes Cbl-dependent polyubiquitination and proteosomal degradation.^14^ On polyubiquitination of targets, Cbl then promotes its own polyubiquitination and subsequent autodegradation.^14^ We observed that Cbl protein from WT/WT PBMCs promoted robust polyubiquitination of EGFR, whereas Cbl from WT/c.1110_1112del PBMC (from B-III-1 and B-III-2) showed diminished capacity to polyubiquitinate EGFR (figure 1I). Similar results were observed for A-II-3. These results suggested that the heterozygous *CBL* mutations we detected in our patients resulted in loss of Cbl-mediated ubiquitination activity.

### Cbl-regulated cell surface receptor degradation and MAPK signaling in PBMCs from patients with heterozygous CBL variants

Given the differences we observed in the ubiquitination capacity of PBMCs from our patients, we next explored whether there was reduced cellular degradation of the Cbl-regulated cell surface receptor EGFR and the potential impact of that on downstream EGFR-mediated MAPK signaling. After 120 minutes of treatment with epidermal growth factor (EGF; a growth factor that stimulates the EGFR), we observed that relative EGFR expression and MAPK phosphorylation in patient-derived PBMCs were increased compared with healthy control cells (*p* = 0.03 and *p* = 0.008; figure 1, J and K). These results suggested aberrant growth factor receptor degradation and signaling associated with mutant Cbl in PBMCs from patients with heterozygous *CBL* mutations and cerebral arteriopathy.

### siRNA-mediated silencing of CBL in endothelial cells: effects on VEGFR-2–induced tyrosine kinase signaling and angiogenesis

**Figure 3:**
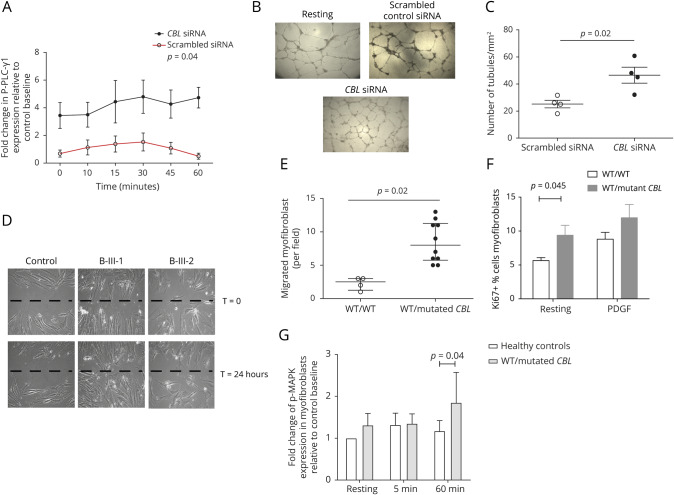
Enhanced angiogenesis in siRNA CBL-transfected endothelial cells and enhanced proliferation, migration, and PDFGR-induced tyrosine kinase signaling in smooth muscle cells (SMCs) from patients with heterozygous CBL mutation (A) Human umbilical endothelial vein cells (HUVECs) were transfected with siRNA targeting *CBL* or a scrambled siRNA control and then stimulated with 20 ng/mL VEGF for the indicated periods of time before staining to explore expression of phosphorylated PLC-γ1. Silencing of *CBL* resulted in significant upregulation of relative phosphorylated-PLC-γ1 expression in siRNA *CBL* transfected cells compared with control scrambled transfected HUVECs, *p* = 0.04. (B) Representative images of HUVEC capillary network formation on matrigel for resting endothelial cells, scrambled control siRNA, and *CBL* siRNA-transfected endothelial cells. Five independent fields were assessed for each well and the mean numbers of tube branches determined. (C) There was enhanced HUVEC capillary network formation in a matrigel assay in siRNA *CBL-*transfected cells compared with scrambled control cells, *p* = 0.02. (D and E) Human dermal fibroblast cells (HDFCs) from patients with heterozygous *CBL* mutations were treated with 5 ng/mL transforming growth factor 1 for 14 days to induce trans-differentiation into SMCs. A standard scratch assay was then performed on cultured monolayer of SMCs derived from HDFCs of healthy controls and patients with *CBL* mutations. There was an increase in migration of SMC patients with heterozygous CBL mutations compared with control WT/WT cells, *p* = 0.02. (F) Patient-derived SMC-like cells/myofibroblasts also exhibited enhanced proliferation when compared with control cells, *p* = 0.045. (G) SMC-like cells/myofibroblasts were stimulated with 20 ng/mL PDGF for the indicated periods of time before staining to explore changes in phosphorylated MAPK expression. There was enhanced phosphorylation of MAPK at 60 minutes induced by PDGF receptor in patient-derived SMCs compared with control cells, *p* = 0.04. Data were expressed as fold change relative to mean baseline expression for control and plotted as mean of triplicate samples ± SEM. *p* < 0.05 calculated by analysis of variance and unpaired *t* tests were considered significant. CBL = casitas B-lineage lymphoma; MAPK = mitogen-activated protein kinase; PDGF = platelet-derived growth factor.

We next explored whether defective degradation of Cbl-regulated cell surface receptors enhances tyrosine kinase signaling in endothelial cells, thus promoting angiogenesis and contributing to the moyamoya capillary pattern we observed in our patients. It is well established that endothelial cell activation of the signaling effector PLC-γ1 through VEGF receptor 2 (VEGFR-2) is in part responsible for angiogenesis in vivo.^15,16^ Cbl E3 ubiquitin ligase constitutively associates with PLC-γ1 via its C-terminal domain and conditionally interacts with VEGFR-2 via the N-terminal/TKB domain, acting as a negative regulator of the signal transduction pathway that controls angiogenesis.^15,16^ We therefore examined whether there was attenuation of VEGFR-2 induced tyrosine kinase signaling in siRNA *CBL-*transfected HUVECs compared with scrambled control endothelial cells. Silencing of *CBL* by siRNA in HUVECs resulted in significant upregulation of the relative expression for the VEGFR-2–induced signaling effector PLC-γ1 compared with control cells, *p* = 0.04 (figure 3A) and also enhanced HUVEC capillary network formation in matrigel (difference between means 21.29 ± 6.427, 95% CI 5.3–27, *p* = 0.02; figure 3, B and C). These results indicated that silencing *CBL* enhances VEGFR-2–induced signal transduction in endothelial cells, promoting angiogenesis.

### PDFGR-induced tyrosine kinase signaling and SMC myofibroblast proliferation and migration in patients with heterozygous CBL variants

The vascular pathology observed in several other genetic cerebral arteriopathies relates to increased proliferation and migration of SMCs and myofibroblasts to the medial layer of the cerebral arterial wall that subsequently causes arterial occlusion.^17^ We thus also sought to determine whether aberrant degradation of Cbl-regulated surface receptors associated with mutant *CBL* enhanced cellular signaling pathways and promoted fibroblast and myofibroblast proliferation and migration.

Dermal fibroblasts directly explanted from patients (A-II-2, B-III-1, B-III-2, and B-II-2) were exposed to transforming growth factor 1 and trans-differentiated into SMCs. These cells exhibited enhanced migration (difference between means 6.2 ± 1.595, 95% CI 2.774–9.776, *p* = 0.02; figure 3, D and E), increased proliferation (difference between means 3.14 ± 1.087, 95% CI 0.1228–6.157, *p* = 0.045; figure 3F), and upregulation of MAPK expression compared with control cells (difference between means 0.65 ± 0.441, 95% CI −0.573 to 1.875, *p* = 0.04; figure 3G). siRNA silencing of *CBL* had a similar effect on the migratory capacity of HBVAF (difference between means 3.283 ± 1.544, 95% CI 0.03–6.5, *p* = 0.04), proliferation (difference between means 1.907 ± 0.07, 95% CI 1.691–2.123, *p* = 0.0001), and for phosphorylated MAPK expression (difference between means 0.884 ± 0.4778, 95% CI −0.4425 to 2.21, *p* = 0.01). Therefore, these results suggested that mutant *Cbl* enhances PDFGR-induced tyrosine kinase signaling and promotes SMC proliferation and migration in patients with heterozygous variants in *CBL* and cerebral arteriopathy.

### Inhibition of the RAF-RAS-MAPK pathway: effects on the migratory and proliferative capacity of fibroblasts derived from patients with heterozygous CBL variants

**Figure 4:**
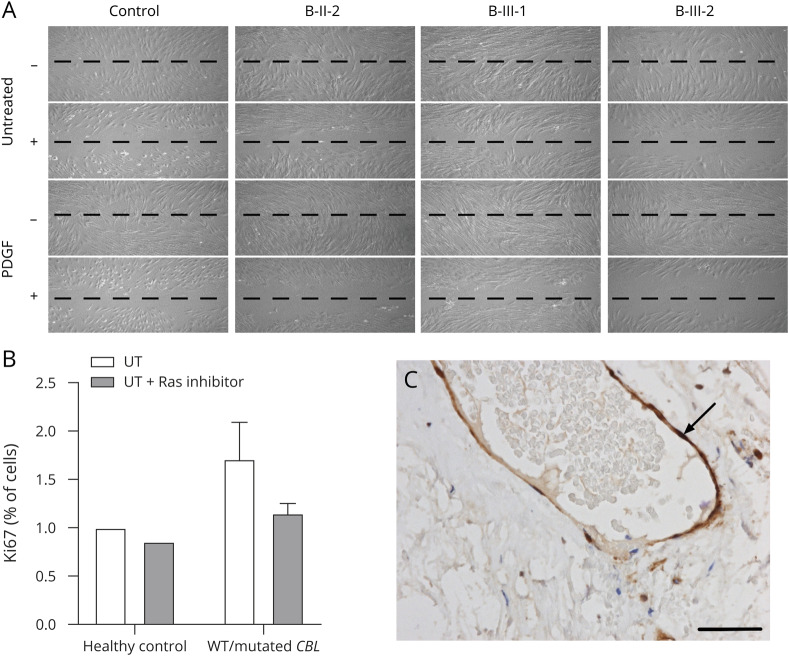
Inhibition of the RAF-RAS-MAPK pathway supresses the migratory and proliferative capacity of fibroblasts derived from patients with heterozygous *CBL* variants (A and B) Resting and PDGF-treated patient-derived human dermal fibroblast cells (HDFCs) explanted from B-II-2, B-III-1, and B-III-2 were treated with 5 μM of sorafenib, a protein kinase inhibitor that targets the RAF-RAS-MAPK pathway. Treatment with sorafenib suppressed the migratory and proliferative capacity of HDFCs derived from patients to levels seen in controls cells. (C) Immunohistochemistry for pERK on dura taken during surgery on patient A-II-3 showed strong endothelial expression (arrow) in dural blood vessels. Scare bar is 50 μm. CBL = casitas B-lineage lymphoma; MAPK = mitogen-activated protein kinase; PDGF = platelet-derived growth factor.

We next examined whether targeting the RAF-RAS-MAPK pathway with sorafenib, a multikinase inhibitor with activity against RAF kinase, suppresses the enhanced proliferative and migratory capacity of HDFCs derived from patients with heterozygous CBL variants. Resting and PDGF-treated patient-derived HDFCs treated with 5 μM of sorafenib exhibited suppression of migratory and proliferative capacity to levels seen in controls cells (figure 4, A and B).

### Arterial histology associated with heterozygous CBL variants

Analysis of the arterial tissue from dural biopsy obtained post–IC-EC procedure for A-II-3 showed positive pERK staining in endothelial cells (figure 4C).

## Discussion

**Figure 5:**
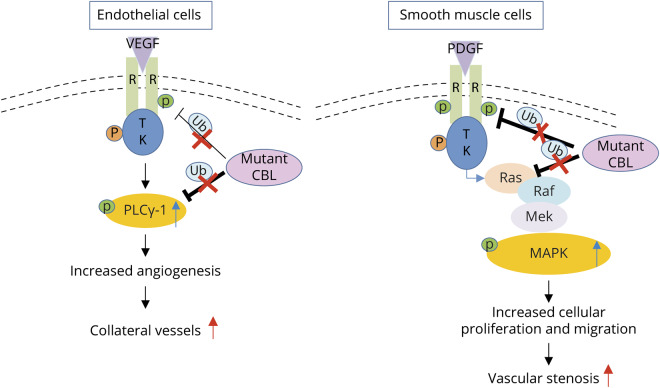
Schematic representation of possible disease mechanisms in arteriopathy associated with mutant CBL Loss of Cbl-mediated ubiquitination activity in mutant *CBL* endothelial and smooth muscle cells results in reduced *CBL*-regulated cell surface receptor degradation and enhanced cellular tyrosine kinase signaling promoting angiogenesis, collateral vessel formation, smooth muscle cell proliferation and migration, and vascular stenosis. CBL = casitas B-lineage lymphoma; MAPK = mitogen-activated protein kinase; P = phosphorylated; PDGF = platelet-derived growth factor; TK = tyrosine kinase; Ub = ubiquitination; VEGF = vascular endothelial growth factor.

We identified heterozygous mutations in *CBL* in several patients presenting with cerebrovascular disease and provide an insight into how mutant Cbl could mechanistically cause this arteriopathy (figure 5). We show that impaired Cbl-mediated degradation of cell surface receptors and dysregulated intracellular signaling through the RAS-MAPK pathway may contribute to the pathogenesis of *CBL-*mediated arteriopathy. Mutated *CBL* failed to control the angiogenic signal relay of VEGFR-2 and led to subsequent prolonged PLC-γ1 activation in endothelial cells, thus enhancing vascular angiogenesis and capillary network formation, and thus possibly contributing to the basal collateral vessel formation we observed. Mutant *CBL* also promoted myofibroblast migration and proliferation via the PDGF receptor pathway, which could contribute to the vascular occlusive disorder observed in patients with heterozygous *CBL* mutations. These effects were abrogated in vitro following treatment with a RAS pathway inhibitor.

Genetic factors have long been suspected in cerebral arteriopathy such as moyamoya arteriopathy, based on increased incidence in certain ethnicities (especially East Asians) and a familial case prevalence of 10%.^18^ For instance, variants of *RNF213* account for the genetic susceptibility of Asian patients, with approximately 80% with moyamoya harboring the heterozygous p.R4810K founder variant.^19^ RNF213 has been shown in vivo and in vitro to play a significant role in controlling angiogenesis through regulating the expression of matrix metalloproteinases in endothelial cells.^20^ We now expand the spectrum of genetic arteriopathies to include *CBL*-mediated arteriopathy. We observed that mutated Cbl significantly inhibited E3 ubiquitin ligase activity and overall led to loss of negative control over the expression of several receptor tyrosine kinases, including EGFR, VEGFR-2, and PDGR, thus enhancing downstream signaling pathways such as the RAS-MAPK pathway. Occlusive vascular disease could arise secondary to this increased cellular proliferation caused by aberrant receptor tyrosine kinase signaling. In line with previous studies, we also show that mutant *CBL* function abrogates downregulation of active PLC-γ1, subsequently increasing angiogenesis.^15,16^ Ubiquitination of PLC-γ1 by *CBL* serves as an inhibitory mechanism to VEGFR-2–induced angiogenesis.^15,21^ This loss of negative control of VEGFR-2–induced angiogenesis could be relevant to the development of the basal collateral network, which characterizes the radiologic moyamoya arteriopathy phenotype. Our findings are also in keeping with previous animal model studies showing that mutant *CBL* enhances angiogenesis in zebrafish.^15^

Analysis of the arterial tissue obtained from A-II-3 showed pERK-positive staining in endothelial cells, providing direct evidence of abnormal signaling of this pathway in affected cerebral vasculature from a patient. Additional evidence for the specificity of our findings comes from the inhibition experiments with sorafenib that specifically targets the RAF-RAS-MAPK signaling pathway. Treatment with this therapeutic agent in vitro suppressed the enhanced migratory and proliferative capacity of fibroblasts obtained from patients with heterozygous *CBL* variants to levels seen in cells obtained from healthy controls. It remains to be established whether treatments targeting this pathway can prevent stroke recurrence in patients with arteriopathy associated with mutant Cbl.

Of interest, previous studies have suggested that germline mutations of *CBL* define predisposition to juvenile myelomonocytic leukemia.^6,7^ None of our patients developed a hematologic malignancy to date. It may be the case that heterozygous *CBL* mutations lead to dysregulation in cell proliferation via the RAS-MAPK pathway, but do not cause malignancy unless reduced to homozygosity in target tissue. This could indicate that a homozygous state or a “two-hit” process is necessary for oncogenicity.

We suggest that clinicians should consider *CBL* screening in patients with moyamoya-like and other systemic arteriopathies, even in the absence of obvious developmental abnormalities or malignant hematologic manifestations suggestive of a typical RASopathy. The radiologic appearances we observed in association with heterozygous *CBL* mutation were of an occlusive arteriopathy with extensive basal collaterals, unlike the lack of collateral formation seen for instance in *ACTA2*-related arteriopathies.^17^ More atypical and heterogeneous imaging findings could ultimately also be a feature of arteriopathy caused by *CBL* mutation, however, an issue that will become clearer as more patients are described. Thus, the contribution of *CBL* mutations to other occlusive cerebral arteriopathies and arterial ischemic stroke remains to be established in future prospective studies of genetic screening in larger cohorts of unselected cases.

Although all affected patients had cerebral arteriopathy, we acknowledge the heterogeneity of presentation in the cohort of patients described herein. It is possible that additional mechanisms promoting fibroblast proliferation and migration, angiogenesis, and collateral formation are involved in *CBL*-mediated arteriopathy. The specificity of our proposed mechanism for *CBL*-mediated cerebral arteriopathy needs to be established in larger cohorts, including repeating similar biological assays in cases with a similar radiologic cerebral arteriopathy phenotype but with no *CBL* variants identified. Genotype-phenotype correlations were not possible in this cohort, but may become apparent as more cases are described. None of the patients had evidence of a systemic arteriopathy on initial screening, but vascular surveillance is ongoing in light of previous reports suggesting that several genetic arteriopathies causing stroke are not just confined in the CNS and may be more widespread than previously considered.^17,22^

In summary, we describe monoallelic variants in *CBL* in patients from 3 unrelated kindreds as the likely cause of cerebral arteriopathy. We provide a possible mechanism for the observed arteriopathy through impaired *CBL*-mediated degradation of cell surface receptors and dysregulated intracellular RAS signaling. Future exploration of this pathway could facilitate identification of novel or repurposed druggable targets for cerebral arteriopathy.

## Glossary

*CBL*: casitas B-lineage lymphoma
CI: confidence interval
EGFR: epidermal growth factor receptor
HBVAF: human brain vascular adventitial fibroblast
HDFC: human dermal fibroblast cell
HUVEC: human umbilical vein endothelial cell
ICA: internal carotid artery
MAPK: mitogen-activated protein kinase
MCA: middle cerebral artery
PBMC: peripheral blood mononuclear cell
PDGF: platelet-derived growth factor
PLC-γ1: phospholipase C-γ1
SMC: smooth muscle cell
VEGF: vascular endothelial growth factor
VEGFR-2: VEGF receptor 2
WES: whole-exome sequencing
